# Properties of Rigid Polyurethane Foam Modified by Tung Oil-Based Polyol and Flame-Retardant Particles

**DOI:** 10.3390/polym12010119

**Published:** 2020-01-05

**Authors:** Wei Zhou, Shu-Jie Hao, Guo-Dong Feng, Pu-You Jia, Xiao-Li Ren, Meng Zhang, Yong-Hong Zhou

**Affiliations:** 1 Institute of Chemical Industry of Forestry Products, Chinese Academy of Forestry, Nanjing 210042, China; vvzhou90@163.com (W.Z.); zyh@icifp.cn (Y.-H.Z.); 2 Co-Innovation Center of Efficient Processing and Utilization of Forest Resources, Nanjing Forestry University, Nanjing 210037, China; 3 Key Lab of Biomass Energy and Materials, Jiangsu Province, Nanjing 210042, China; 4 Key Lab of Forest Chemical Engineering, SFA, Nanjing 210042, China

**Keywords:** tung oil, polyol, catalyst-free, flame retardant particles, rigid polyurethane foam

## Abstract

Although tung oil is renewable, with an abundant production and low price in China, and it is used to synthesize different polyols for rigid polyurethane foam (RPUF), it remains a challenge to improve the properties of RPUF by redesigning the formula. Therefore, we propose four novel compounds to strengthen the properties of RPUF, such as the catalyst-free synthesis of tung oil-based polyol (PTOK), aluminum phosphate micro-capsule (AM), silica micro-capsule (SiM), and grafted epoxidized monoglyceride of tung oil on the surface of SiO_2_ (SiE), which were designed and introduced into the RPUF. Because of the PTOK with a catalytic function, the foaming process of some RPUF samples was catalyst-free. The results show that the incorporation of AM, SiM, and SiE, respectively, endow RPUF with a better thermal stability at a high temperature, and the *T*_5%_, *T*_max1_, and *T*_max2_ of RPUF appeared to be reduced, however, the *T*_max3_ and residue rate at 800 °C were improved, which may have a positive effect on the extension of the rescue time in case of fire, and the limiting oxygen index (LOI) value was increased to 22.6%. The formula, containing 25% PTOK made the RPUF environment-friendly. The results were obtained by comparing the pore size and mechanical properties of the RPUF—the AM had a better dispersion in the foam, and the foam obtained a better mechanical, thermal, and flame retardancy.

## 1. Introduction

Polyurethane (PU) is a kind of compound with a polyurethane structure, prepared from polyols and polyisocyanate, which is widely used in coatings, adhesives, elastomers, and foams [1,2,3,4]. Traditional polyurethane mainly comes from non-renewable fossil fuels, and tensions in the Middle East also have an impact on the oil price and created an uncertainty of supply internationally [5]. There is the irrefutable fact that the fluctuation of the oil price and the uncertainty of the supply will affect the development of the polyurethane industry, which has caused people to consider petrochemical resources to be replaced by the biomass resources in the manufacturing of PU [6]. Moreover, people have taken the effect on the environment resulting from the PU synthesis, use, and disposal phases into account. Renewable resources could be used for the synthesis of PU, which would not only cut down on the greenhouse effect and, gradually, on serious global environment pollution, but it also has a trend towards development in the coming years [7]. The waste of PU is difficult to degrade in the disposal phases, and it has an adverse effect on the application of PU. Straw, cellulose, starch, lignin, vegetable oils, and other natural macro-molecules can be regarded as green chemical raw materials from renewable resources, because of their abundant yields, cheap price, and biodegradable capacity [8,9,10,11,12]. Hence, a series of studies on biomass utilization for the preparation of PUs have caused extensive and strong concern among researchers from all around the world.

Vegetable oils as a feedstock, instead of crude oil, for the production of PU materials have been extensively studied. The main composition of vegetable oils is called triglyceride, and taking their price into consideration, products such as palm oil, soybean oil, and rapeseed oil are the most affordable for large-scale production. Among PU products, polyurethane foams (PUF), which are divided into soft PUF, rigid polyurethane foam (RPUF), and semi-rigid PUF, have been widely used used in various fields of auto-motion, construction, furniture, aerospace, and packaging [13,14]. RPUF is widely used in the acoustic field, and for thermal insulation or as a core material of insulation for sandwich panel application in the construction industry [15]. With the expansion of the applications of RPUF, the demand for their properties of is much higher. For example, the thermal insulation material actively promoted by China now requires that RPUFs should not only provide a good thermal insulation performance of thermal conductivity, bulk density, high temperature resistance, compressive strength, linear expansion coefficient, flexural strength, and pH value [16,17,18,19,20,21], but they also need to have environmental protection and flame-retardant performances [22]. However, RPUF is a polymeric material, which has a cellular structure with a lower density, larger specific surface area, and inflammability. Moreover, researchers are not satisfied with the obtained properties of RPUFs made from vegetable oils, such as their flame retardancy and mechanical properties.

Considering authority, environment, safety, and health factors, halogenated flame retardants should not be regarded as being effective flame retardants of RPUF. Halogen-free flame retardants have advantages of being green and environmentally friendly, and there are no hazardous consequences to human health or the ecological environment with using them. Generally, there are two means to improve the flame resistance of RPUF, including additives and reactive flame retardants. One is the direct addition of flame-retardant agents, such as layered silicate, phosphonium salt, expandable graphite, ammonium polyphosphate, and SiO_2_ [23,24,25,26,27,28]. Another method is to use reactive flame retardants to be involved in the foaming process, which is a part of the structural components of RPUFs. Usually, polyols are modified to be reactive flame-retardant polyols. For example, polyols are modified by phosphorous-, nitrogen-, and silicon-containing compounds [29]. One problem with this is that a small proportion of flame-retardant elements in RPUF play a limited role in flame retardancy. Moreover, it is difficult to prevent the toxic gases produced during the combustion of RPUF; many fire incidents show that most people die from smoke choking and toxic gases. Hence, the smoke suppression and flame retardancy of RPUF must be considered in case of fire danger.

In this work, we combined two methods of modification of RPUFs, that is, structural modification and the addition of a flame retardant. Micro-capsule flame retardants are tiny particles containing a flame-retardant agent surrounded by a shell. Because of the advantage of the trend to form char structures of SiO_2_, SiO_2_ was modified with epoxidized monoglyceride of tung oil (EGTO) in order to obtain grafted epoxidized monoglyceride of tung oil on the surface of SiO_2_ (SiE). Moreover, silica micro-capsules and aluminum phosphate micro-capsules were also synthesized and used as flame retardant additives, which were incorporated into the RPUFs. For example, aluminum phosphate and SiO_2_ were encapsulated with ammonium phosphomolybdate to improve their dispersibility in the mixture of polyols and polyisocyanates. The influences of PTOK and three flame retardant particles on the thermal property, apparent morphology, flame retardancy, and mechanical properties of RPUF were discussed.

## 2. Materials and Methods

### 2.1. Materials

SiO_2_, aluminium phosphate (ALP), ammonium molybdate (AMM), and 3-(2-aminoethylamino) propyldimethoxymethylsilane (KH-602) were purchased from Shanghai Aladdin Industrial Corporation, China. Phosphoric acid, nitric acid, and dibutyltin dilaurate (DBTDL) were purchased from Xilong Chemical Co., Ltd., Shantou, China. Polyether polyols 4110 (PPG4110, the hydroxyl value was 430 mgKOH/g and the viscosity was 2500–4000 mPa∙s at 25 °C) was obtained from Guangzhou Hongna Chemical Co., Ltd., Guangzhou, China. Polyisocyanate (PAPI; the NCO content was 30.3 wt %) was bought from Yantai Wanhua Polyurethane Co., Ltd., China. The surfactant was from Nanjing Dymatic Shichuang Chemical Co., Ltd., Nanjing, China. Epoxidized monoglyceride of tung oil (EGTO) [30] and deionized water were made in the laboratory. The blowing agent (HFC-365mfc) was obtained from Dongguang Changze Chemical Co., Ltd., Dongguang, China. All of the above materials were used directly.

### 2.2. Synthesis of Aluminum Phosphate Micro-Capsule (AM)

**Figure 1:**
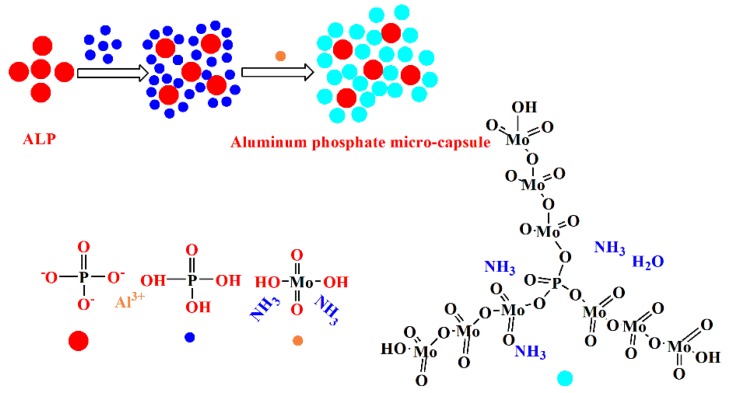
The synthesis route of an aluminum phosphate micro-capsule. ALP—aluminium phosphate.

The synthesis route of the aluminum phosphate micro-capsule is shown in Figure 1, including two steps. Firstly, a mixture of aluminum phosphate (1 g) and 85% phosphoric acid (5 mL) were stirred at room temperature using a magnetic stirrer for 8 h. Then, ammonium molybdate (0.5 g) and nitric acid were added into the suspension liquid to adjust the pH to a value of 1.0, and they were allowed to sit for 24 h. The obtained deposit, namely AM, was filtered, washed several times by using deionized water, and dried in a vacuum in the oven at 60 °C for 24 h.

### 2.3. Synthesis of Silica Micro-Capsule (SiM)

The synthesis route of the silica micro-capsule, which is similar to that of the aluminum phosphate micro-capsule, including two steps, is as follows. Firstly, the mixture of SiO_2_ (1 g) and 85% phosphoric acid (10 mL) was stirred at room temperature using a magnetic stirrer for 8 h. Then, ammonium molybdate (0.5 g) and nitric acid were added into the suspension liquid to adjust the pH to a value of 1.0. The above-mentioned mixture was allowed to sit for 24 h. Finally, the deposit was found to be a product of SiM, which was obtained by filtration. It was washed several times using deionized water and was dried in a vacuum in the oven at 60 °C for 24 h.

### 2.4. Synthesis of Grafted Epoxidized Monoglyceride of Tung Oil on the Surface of SiO_2_ (SiE)

**Figure 2:**
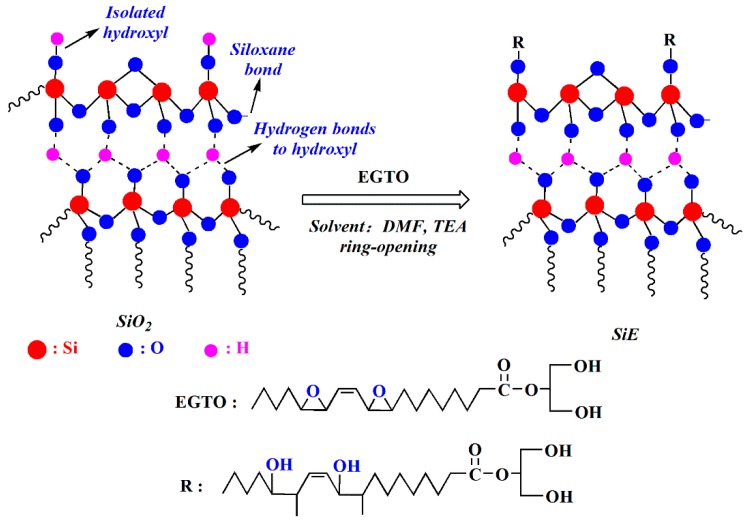
The synthesis route of grafted epoxidized monoglyceride of tung oil on the surface of SiO_2_ (SiE). DMF—*N*,*N*-dimethylformamide; TEA—triethylamine; EGTO—epoxidized monoglyceride of tung oil.

The preparation of epoxidized monoglyceride of tung oil (EGTO) was used based on the literature [30]. SiO_2_ (2.21 g) and EGTO (24 g) were dissolved in 150 mL *N*,*N*-dimethylformamide (DMF) and 0.5 mL triethylamine (TEA). The reaction was done under a temperature of 120 °C, and was mechanically stirred for 20 h under N_2_ protection. After the reaction was completed, the product was washed with tetrahydrofuran (THF), and the collected precipitate was dried in a vacuum in the oven at 60 °C for 24 h. The synthesis route of SiE is shown in Figure 2.

### 2.5. Synthesis of Tung Oil-Based Polyol (PTOK) Modified by KH-602

**Figure 3:**
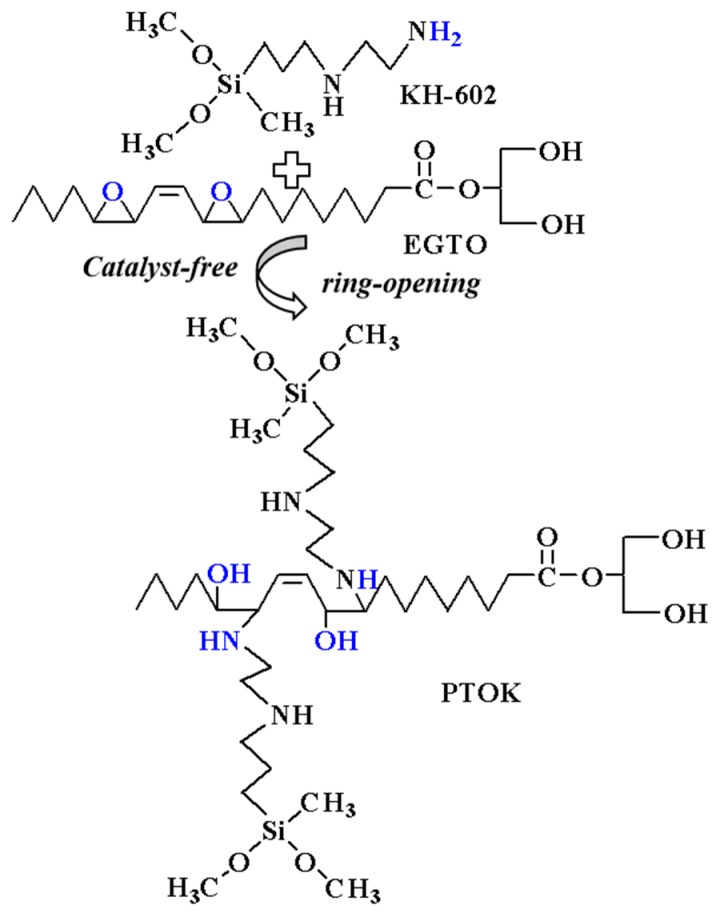
The synthesis route of tung oil-based polyol (PTOK).

The synthesis route of PTOK is shown in Figure 3. It was prepared by the ring opening reaction of EGTO (100 g) and KH-602 (7.94 g) under N_2_ with vigorous stirring for 2 h at 70 °C. It was kept nearly catalyst-free to ensure that the primary amino group of KH-602 would react with the epoxy group, while the secondary amine group hardly did so. The obtained brown liquid was the product, PTOK. The hydroxyl value and the viscosity of PTOK were 344.71 mgKOH/g and 3.81 Pa∙s (25 °C), respectively.

### 2.6. RPUF Synthesis

Some RPUF samples were produced by a one-step process without a catalyst, because the PTOK possessed the property of auto-catalysis. The foam formation is shown in Table 1. It was found that when the PTOK content was higher than 25%, the mixture could hardly be mixed evenly, so it was kept at 25%. AM, SiM, SiE, PTOK, and PPG4110 were composed of complex polyols. Meanwhile, the foam stabilizer, foaming agent, and water were mixed together to get a homogeneous mixture. Then, PAPI was added into the viscous mixture quickly, and the mixture under strong agitation. After the foam was completely cured, it was placed in a drying oven at 60 °C for 12 h.

### 2.7. Characterization

The FTIR spectra of the sample was recorded using a Nicolet (iS10, Waltham, MA, USA) FTIR spectrometer, with a wavenumber number range of 4000 to 500 cm^−1^, at a 4 cm^−1^ resolution and with 32 scans. A Malvern Mastersizer 2000 laser particle diameter instrument (Malvern Instruments, Malvern, UK) was used to investigate the particle size of the AM, SiM, and SiE, respectively. The micro-structures of the flame-retardant particles and RPUF were recorded using SEM (S3400N, Hitachi, Tokyo, Japan), respectively. The limiting oxygen index (LOI) of the RPUF was tested using the GB/T 2406 oxygen index meter (TESTECH, Suzhou, China). The test was measured according to ASTM D2863. The dimensions of the RPUF were 100 × 10 × 10 mm. The amount of sample was studied using thermogravimetric analysis (TGA) with a NETZSCH TG 209F3 (Gebruder-Netzsch-Straβe, Selb, Germany), under a stream of N_2_, with the temperature increasing at 10 °C/min from 40 °C to 800 °C. The thermal conductivity test was as follows: the RPUF was tested using a thermal conductivity analyzer (JB-DZDR-P, Shanghai, China), according to ASTM C518, and the precision of the RPUF was 50 × 50 × 20 mm. The density of the RPUF was detected according to ASTM D1622. The size of the RPUF was 50 mm × 50 mm × 50 mm. Each set of samples consisted of five samples, and the final result was obtained from the average value. The mechanical test of RPUF was conducted on a universal testing machine (CMT4000, Shenzhen, China). The compression test was as follows: the dimensions of the RPUF were 50 × 50 × 50 mm, which was compressed to a 10% strain with speed rate of 5 mm/min. The tensile test was as follows: the dimension of RPUF was 20 × 25 × 20 mm, which was stretched until fractured, under a speed of 2 mm/min. The bending test was as follows: using an RPUF with dimensions of 12 × 25 × 20 mm.

## 3. Results and Discussion

### 3.1. Chemical Modification

**Figure 4:**
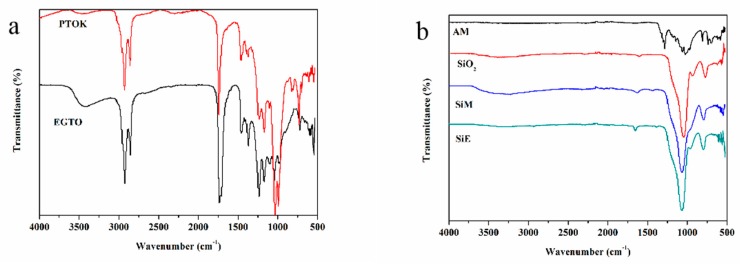
FTIR of PTOK, EGTO (**a**), AM, SiO_2_, SiM, and SiE (**b**).

A very broad peak in the range of 3200 to 3600 cm^−1^ (Figure 4a) belonged to the hydroxyl group of EGTO and PTOK. In the FTIR of EGTO, the peaks at 2860 and 2920 cm^−1^ were ascribed to stretching the vibration absorption of methyl and methylene; moreover, the peaks at 1460 and 1370 cm^−1^ corresponded to bending the vibration absorption of methyl and methylene, respectively. The characteristic absorption peak at 1730 cm^−1^ was ascribed to the ester group; 1240, 1170 and 1100 cm^−1^ were related to the ether bond of EGTO; 1050 cm^−1^ was attributed to the C–O–C group; and 908 cm^−1^ was ascribed to the epoxy group [31]. While referring to the FTIR spectrum of PTOK, the different peaks demonstrated the presence of an -NH group (3440 cm^−1^), Si–O bond (987 and 823 cm^−1^), and Si–C bond (731 and 693 cm^−1^). These results indicated that KH-602 was successfully incorporated into the structure of EGTO.

The band centered at 3184 cm^−1^, observed in the FTIR spectrum of AM, was characteristic of the vibration *V_N-H_* NH_4_^+^, which confirmed the formation of AM by ammonium molybdate and aluminum phosphate. Strong peaks were observed at about 1305 and 1283 cm^−1^ from the P=O of the AM. The characteristic peaks at 1060 and 1027 cm^−1^ corresponded to the P–O of AM; all of the above results confirmed that the AM was successfully synthesized. Furthermore, a peak appeared at 3328 cm^−1^ belonging to the hydroxyl group on the surface of the silica in the spectrum of SiO_2_. New bonds observed in the spectrum of SiM were completely different from the bonds of NH_4_^+^ observed at the peak of 3226 cm^−1^. The bond corresponded to the stretching vibration absorption of Si–O at 1065 cm^−1^. These important results of the bonds suggest that the SiM was prepared successfully. In the spectra of the SiE, the absorption peak at 3253 cm^−1^ was attributed to the –OH group in the SiE. The absorption peak at 1065 cm^−1^ was assigned to an ether bond (C–O), and the absorption peaks at 960 and 795 cm^−1^ were related to the silanol group and Si–C, respectively. All of these occurrences were indicative of the successful grafted EGTO on the surface of SiO_2_.

### 3.2. Particle Size Analysis and Morphology of Flame-Retardant Particles

**Figure 5:**
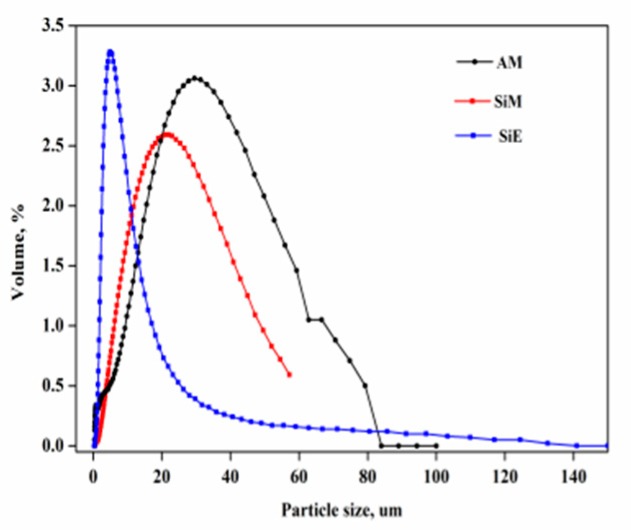
Diameter distribution of AM, SiM, and SiE.

The basic size data of AM, SiM, and SiE was given in Table 2. Obviously, the relation between the specific surface area and the ration of the average particle size of AM, SiM and SiE was: SiE_SSA_ > AM_SSA_ > SiM_SSA_, AM_D50_ > SiM_D50_ > SiE_D50_, respectively. Figure 5 showed the diameter distribution curves of three particles. It was observed that the diameter of AM particle was mainly concentrated in the range from 30 to 95 μm, accounting for 67.51%. The diameter of SiM particle was mainly concentrated in the range from 10 to 40 μm, accounting for 68.69%. SiE particle diameter was mainly concentrated in the range from 2.5 to 11 μm, accounting for 69%.

**Figure 6:**
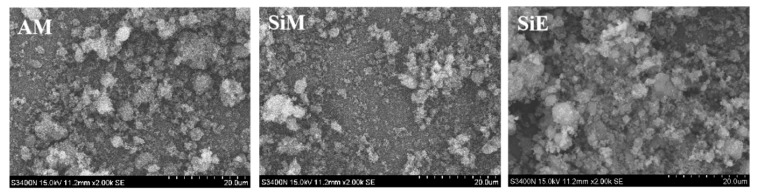
SEM images of AM, SiM, and SiE.

Figure 6 shows micrographs of the three particles; the results of the particle size relationship between AM, SiM, and SiE are consistent with the previous analysis results shown in Figure 5. Meanwhile, the surfaces of the two micro-capsules of AM and SiM were rough and they were scattered, while the surface of SiE was smooth and it agglomerate. When the flame-retardant particles were at work while the rigid polyurethane foam was burning, the agglomerated particles with adverse effects on the foam morphology, flame retardancy and mechanical properties.

### 3.3. Microstructure of RPUF

**Figure 7:**
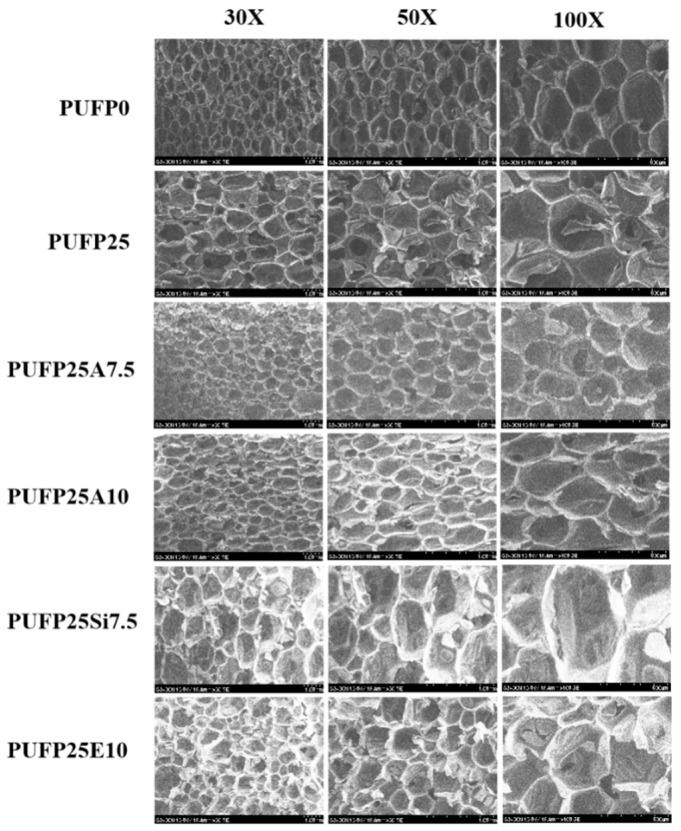
SEM images of RPUF in different magnifications.

To investigate the successful introduction of different flame-retardant particles such as AM, SiM, and SiE into RPUF and the rough surface of RPUF, its typical structure, which is closed cells of a polygonal shape, were observed by SEM (Figure 7). The image of the RPUF taken with different magnifications shows that the foam sustained a closed cell structure in three dimensions, where two adjoining cells shared a wall and three cell edges meet at a curved triangular plateau, the borders from which most of the stiffness and strength of the cell structure were derived [32]. The pore size distribution and structure had an influence on the acoustic performances. In order to systematically study the effect of the particle size and volume fraction of the flame-retardant particles on the cell structure, the flame retardant particles were added into the RPUF at different levels.

As presented in Figure 7, the pores of PUFP25 were larger than those of PUFP0. This phenomenon was caused by the activity of PTOK, which was higher than that of PPG4110, and it was observed that the reaction activity of the amine group with PAPI was higher than those of the other active hydrogen compounds. The produced carbon dioxide contributed to form porous cells [33], and when the carbon dioxide production rate was faster, the cells of the foam were bigger. When AM, SiM, and SiE were introduced into the RPUF, the pores of these composite RPUF samples were greater than those of PUFP0, while were less than those of PUFP25. It can be seen that the cell size of RPUF with different sizes was PUFP25Si7.5 > PUFP25E7.5 > PUFP25A7.5, and the cell size of the RPUF increased with the increase of the particle concentration of the AM. The flame-retardant particles (aggregates or agglomerates) were not seen in the SEM because of the large difference in the size of the cells. By observing the foam structure of RPUF, it was observed that the holes of RPUF with a higher density were spherical, while those of RPUF with a lower density were polyhedral. Spherical and polyhedral shapes were the two basic shapes of the RPUF, and in this study, the structure of the foam was polyhedral.

### 3.4. Flame Retardancy

**Figure 8:**
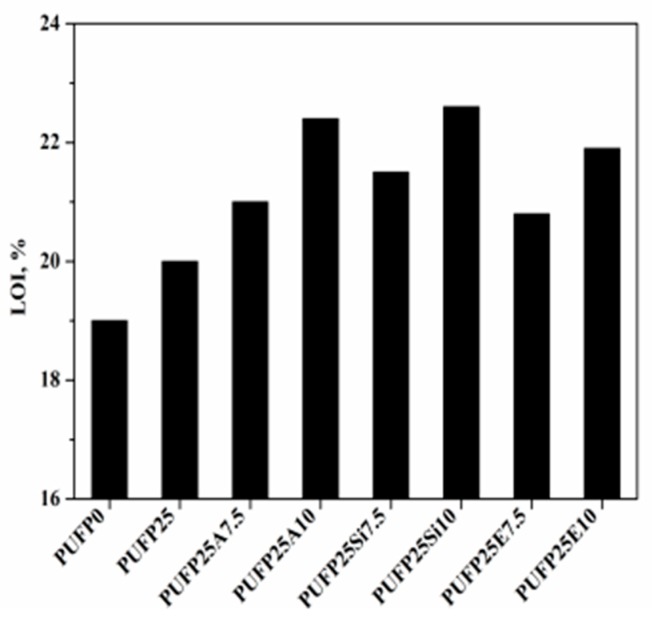
LOI value of RPUF.

The LOI value of the RPUF is presented in Figure 8. Incorporating the flame-retardant particles into RPUF can produce a few flame-retardant chemicals to serve as a protective layer to hold back the diffusion of heat, energy, and O_2_. As a result, RPUF achieved a higher LOI value of 22.6%. In addition, the LOI value of the RPUF increased with both the PTOK proportion and the flame-retardant particles. By contrast, the neat RPUF (PUFP0) only possessed a LOI value of only 19.0%. This phenomenon revealed that the flame-retardant particles (AM, SiM, and SiE) containing at least one or two of these elements, such as molybdenum, phosphorous, silicon, and nitrogen, enhanced the retardancy of the RPUF based on the gas phase, liquid phase, and solid phase flame retardant mechanisms.

### 3.5. Thermal Property

**Figure 9:**
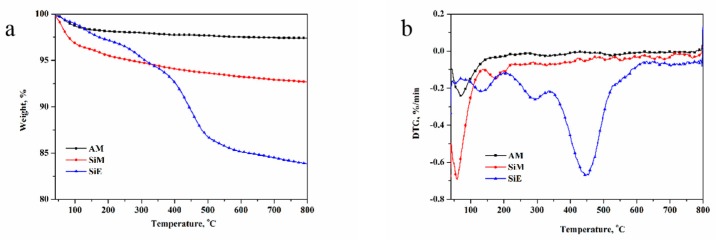
Thermogravimetric (TG) (**a**) and DTG curves (**b**) of AM, SiM, and SiE.

The thermal stability of the sample was investigated using TGA. As can be seen in Figure 9, the temperatures of the flame-retardant particles (AM, SiM, and SiE) corresponding to 2% of the mass loss (*T*_2%_) were 281.55, 71.55, and 144.05 °C, respectively. The residue rate at 800 °C of AM was 97.41%, the residue rates at 800 °C of the other two flame retardant particles of SiM and SiE were 92.68% and 83.89%, respectively. Based on the *T*_2%_ and the residue rate of the flame-retardant particles, AM achieved the best thermal stability, and SiE had a relatively poor thermal stability. This result was attributed to the original thermal stability of ALP, which was better than that of silica, and the residue rate of ALP at 800 °C was 99.63%, while that of silica was only 94.66%. The reason the thermal stability of SiE exhibited a very different weight loss process compared with the other two flame retardant particles was that the structure of EGTO was easily decomposed by heat.

**Figure 10:**
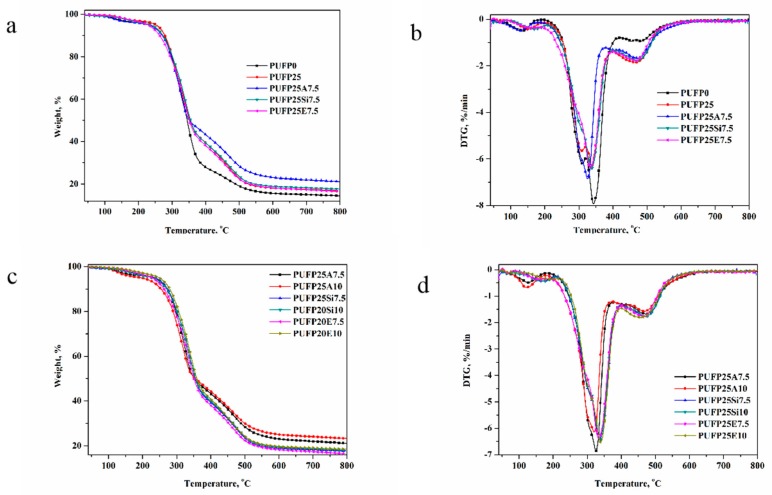
The TG (**a, c**) and DTG (**b, d**) curves of RPUF.

The thermal stability of RPUF was also determined by TGA (Figure 10), and the corresponding thermal degradation data such as initial thermal decomposition temperature (*T*_5%_), maximum decomposition temperature (*T*_max_), and char residue at 800 °C are summarized in Table 3. As can be seen from Table 3, the *T*_5%_ and *T*_max2_ of the other RPUFs were decreased, while the *T*_max3_ and the char residue of the samples at 800 °C were increased with the addition of the PTOK and the flame-retardant particles, except for PUFP0. This is because PTOK introduced into the PU chains will participate in the char formation process of RPUF, and the flame-retardant particles, which were introduced into foam cells, were difficult to decompose substances during the heating process.

In Figure 10, comparing the two-step weight-loss process of another RPUF, such as PUFP0 and PUFP25, shows a different three-step weight-loss process—the first weight-loss steps being from 196.45 to 321.67 °C and 196.45 to 319.32 °C, respectively. This indicates that the introduction of flame retardant particles possessed an effect on the thermal stability of RPUF. In Figure 10c,d, with the increase, all of the RPUF samples were increased, and the *T*_5%_ and *T*_max2_ of RPUF with the addition of SiE showed an increase. This may be attributed to the structure of SiE, which was grafted in polyurethane foam, and it was part of the molecular structure of RPUF. However, the AM and SiM that were added into RPUF only served as particles, without an organic combination with the molecular structure of the foam. The temperature changes of the *T*_max3_ of RPUF with the addition of AM and SiM were slight. While the *T*_max3_ of RPUF decreased with the increase of SiE, this was mainly due to three flame retardant particles, which sustained a higher thermal stability within the scope of the decomposition temperature. This result was also consistent with the previous result about the thermal stability of the AM, SiM, and SiE. The above-mentioned results demonstrated that the RPUF with a better thermal stability at a high temperature, *T*_5%_, *T*_max1_, and *T*_max2_ of RPUF appeared to be reduced; however, the *T*_max3_ and char residue rate were improved, which may play a positive effect on the extension of the rescue time in case of fire.

### 3.6. Mechanical Properties

The PTOK and different flame-retardant particles played a significant role in the mechanical properties, such as the thermal conductivity, compressive strength, tensile strength, and bending strength, of RPUF. Moreover, the density and cell morphology can also contribute to the above mechanical properties.

**Figure 11:**
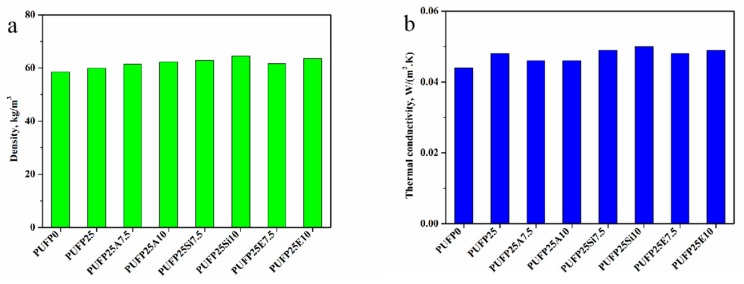
Density (**a**) and thermal conductivity (**b**) of RPUF.

As shown in Figure 11a, with different contents of flame-retardant particles in the RPUF, there was a slight difference in density. The increase of the flame-retardant particles lead to the formation of dense cells, because these flame-retardant particles were evenly filled in the barrier layer surrounding the cell walls or pores of the RPUF. Thus, as the flame-retardant particle content increased, the density also increased slightly from 58.55 kg/m^3^ of PUFP0 to 63.61 kg/m^3^ of PUFP25E10. The thermal conductivity of the foam was related to the density and the thermal conductivity of the gas, such as different blowing agents. Therefore, as the density of each sample was different, and the difference was small, the results of the thermal conductivity of the sample were relatively close.

**Figure 12:**
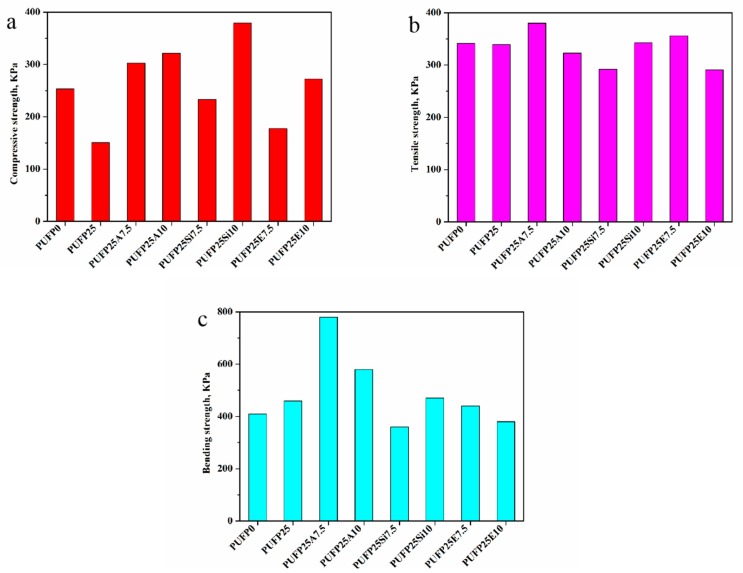
Compressive strength (**a**), tensile strength (**b**), and bending strength (**c**) of RPUF.

The results of the compressive strength, tensile strength, and bending strength are shown in Figure 12. As shown in Figure 12a, the compressive strength of PUFP0 was 253.6 kPa. When the substitution rate of PPG4110 by PTOK was 25%, the compressive strength of PUFP25 was 150.9 kPa. The compression failure was caused by the yield stress of the foam cell edges; with the increase of the pore diameter of PUFP25, the compressive strength of the foam decreased, and this result was consistent in the of SEMs of PUFP0 and PUFP25.

When three flame retardant particles at different levels were incorporated into the RPUF on the basis of 25% PTOK, the same amount of flame-retardant particles, of 7.5 g, was added. Comparing the compression strength of PUFP0 and PUFP25, the increase in the compressive strength of PUFP25A7.5 was the most, and that of PUFP25E7.5 was the second highest. When the addition of particles increased to 10 g, it was observed that the compressive strength of PUFP25Si10 obtained a maximum value of 379.1 kPa. The compression strength of PUFP25A10 was followed by that of PUFP25E10, whose value was 272.5 kPa; the compression strength of RPUF increased with the increase of density. RPUF was quite brittle in tensile testing, and the tensile failure of RPUF was basically brittle failure.

Regarding the result in Figure 12b, it was observed that the tensile strength of the RPUF of PUFP25A10 and PUFP25E10 decreased after the increased content of the flame-retardant particles, which were 323 and 291 kPa, respectively. Only when the SiE was added, was the tensile strength increased. Comparing PUFP0 and PUFP25, where the contents of the flame-retardant particles were 0, it was seen that the tensile strength of the samples, except for PUFP25A10 and PUFP25SiE10, were reinforced by adding the different contents of flame-retardant particles into the RPUF.

It can be observed from Figure 12c that the bending strength of PUFP0 was lower than that of PUFP25. The bending strength of RPUF, which possessed the addition of flame-retardant particles, showed a different trend. Among the bending strengths of all of the RPUF samples, the bending strength of PUFP25A7.5 increased greatly, and when the particle content increased to 10 g, the bending strength decreased. The bending strength of PUFP25Si7.5 was lower than those of PUFP0 and PUFP25. When the content of SiM reached 10 g, the bending strength was increased. The change in SiE content had little effect on the bending strength of RPUF, which was similar to that of PUFP0 and PUFP25.

## 4. Conclusions

A novel method was developed to introduce tung oil-based polyol (PTOK) and different flame-retardant particles into RPUFs. The utilization of a renewable resource (tung oil) in RPUF may make RPUF environment-friendly and help us to decrease the dependence on the fossil resources of the synthesis of RPUF to a certain degree. The catalyst-free synthesis of PTOK was used to make the group of primary amines completely take part in the reaction, while the secondary amine group hardly did so. The foaming process of the RPUF samples, except for PUFP0, was catalyst-free because of the PTOK with a catalytic performance. AM possessed a good thermal stability, and the RPUF with AM possessed a high LOI value. SiE had the smallest size and the lowest thermal stability, which caused the flame retardancy and mechanical performance of PUFP25E7.5 and PUFP25E10 to be poor. What is more, it was observed that the bubble hole was bigger, because SiE was produced by EGTO grafted on the surface of SiO_2_, which was a combination of inorganic and organic matter, ans which possessed a higher activity out of the three flame retardants.

## Figures and Tables

**Table 1:** Formation of RPUF ^a^.

RPUF	PTOK (g)	PPG4110 (g)	DBTDL (g)	AM (g)	SiM (g)	SiE (g)
PUFP0	0	100	0.15	0	0	0
PUFP25	25	75	0	0	0	0
PUFP25A7.5	25	75	0	7.5	0	0
PUFP25A10	25	75	0	10	0	0
PUFP25Si7.5	25	75	0	0	7.5	0
PUFP25Si10	25	75	0	0	10	0
PUFP25E7.5	25	75	0	0	0	7.5
PUFP25E10	25	75	0	0	0	10

^a^: every rigid polyurethane foam (RPUF) contained polyisocyanate (PAPI; 118 g), foam stabilizer (3.75 g), foaming agent (25 g), and H_2_O (0.50 g). PPG4110—polyether polyols 4110; DBTDL—dibutyltin dilaurate; AM—aluminum phosphate micro-capsule; SiM—silica micro-capsule.

**Table 2:** Specific surface area and D (50) of AM, SiM, and SiE.

Sample	SSA ^a^, m^2^∙g^−1^	D (50), μm
AM	1.01	51.50
SiM	0.56	17.28
SiE	1.40	5.57

SSA ^a^—specific surface area.

**Table 3:** TG data of RPUF ^a^.

RPUF	*T*_5%_ (°C)	*T*_max1_ (°C)	*T*_max2_ (°C)	*T*_max3_ (°C)	Residue (800 °C, wt %)
PUFP0	256.7	309.6	343.4	456.2	14.52
PUFP25	254.7	311.2	340.8	468.9	16.94
PUFP25A7.5	231.6	−	325.4	471.8	21.18
PUFP27A10	194.0	−	320.0	467.6	23.32
PUFP25Si7.5	236.6	−	338.8	470.4	17.66
PUFP25Si10	226.5	−	337.4	471.9	17.98
PUFP25E7.5	229.1	−	336.8	470.1	16.56
PUFP25E10	249.0	−	340.0	458.0	18.53

^a^: RPUF without the value of *T*_max1_.
